# HMGA1 augments palbociclib efficacy via PI3K/mTOR signaling in intrahepatic cholangiocarcinoma

**DOI:** 10.1186/s40364-023-00473-w

**Published:** 2023-03-29

**Authors:** Zhipeng Li, Huaxin Zhou, Zhijia Xia, Tong Xia, Gang Du, Strohmer Dorothee Franziska, Xiaoming Li, Xiangyu Zhai, Bin Jin

**Affiliations:** 1 Department of Hepatobiliary Surgery, The Second Hospital of Shandong University, Jinan, China; 2 The Second Clinical College of Shandong University, Jinan, China; 3 Department of General, Visceral, and Transplant Surgery, Ludwig-Maximilians-University Munich, Munich, Germany; 4 Organ Transplant Department, Qilu Hospital of Shandong University, Jinan, China

**Keywords:** Intrahepatic cholangiocarcinoma (iCCA), HMGA1, PI3K/mTOR, CDK4/6, Palbociclib, PF-04691502

## Abstract

**Background:**

Intrahepatic cholangiocarcinoma (iCCA) is a highly aggressive cancer that is challenging to diagnose at an early stage. Despite recent advances in combination chemotherapy, drug resistance limits the therapeutic value of this regimen. iCCA reportedly harbors high HMGA1 expression and pathway alterations, especially hyperactivation of the CCND1/CDK4/CDK6 and PI3K signaling pathway. In this study, we explored the potential of targeting CDK4/6 and PI3K inhibition to treat iCCA.

**Methods:**

The significance of HMGA1 in iCCA was investigated with in vitro/vivo experiments. Western blot, qPCR, dual-luciferase reporter and immunofluorescence assays were performed to examine the mechanism of HMGA1 induced CCND1 expression. CCK-8, western blot, transwell, 3D sphere formation and colony formation assays were conducted to predict the potential role of CDK4/6 inhibitors PI3K/mTOR inhibitors in iCCA treatment. Xenograft mouse models were also used to determine the efficacy of combination treatment strategies related to HMGA1 in iCCA.

**Results:**

HMGA1 promoted the proliferation, epithelial-mesenchymaltransition (EMT), metastasis and stemness of iCCA. In vitro studies showed that HMGA1 induced CCND1 expression via promoting CCND1 transcription and activating the PI3K signaling pathway. Palbociclib(CDK4/6 inhibitor) could suppress iCCA proliferation, migration and invasion, especially during the first 3 days. Although there was more stable attenuation of growth in the HIBEpic model, we observed substantial outgrowth in each hepatobiliary cancer cell model. PF-04691502(PI3K/mTOR inhibitor) exhibited similar effects to palbociclib. Compared with monotherapy, the combination retained effective inhibition for iCCA through the more potent and steady inhibition of CCND1, CDK4/6 and PI3K pathway. Furthermore, more significant inhibition of the common downstream signaling pathways is observed with the combination compared to monotherapy.

**Conclusions:**

Our study reveals the potential therapeutic role of dual inhibition of CDK4/6 and PI3K/mTOR pathways in iCCA, and proposes a new paradigm for the clinical treatment of iCCA.

**Supplementary Information:**

The online version contains supplementary material available at 10.1186/s40364-023-00473-w.

## Background

Cholangiocarcinoma (CCA) is a highly aggressive cancer that arises from the biliary epithelial cells and is the second most common primary liver [1]. CCA can be grouped into three anatomic subtypes: intrahepatic CCA (iCCA), perihilar CCA (pCCA), and distal CCA (dCCA)[2].

iCCA comprises about 20% of all CCAs and has recently received considerable attention [3]. A delayed clinical diagnosis of iCCA results in a poor prognosis, especially for those who fail to receive timely surgical treatment, with a 5-year overall survival rate (OS) of 3%[4] and a median survival time of patients less than 12 [5]. Despite recent advances in combination chemotherapy, drug resistance limits the therapeutic value of this regimen. Thus, novel targeted therapies are urgently needed to improve the prognosis of this patient population. High mobility group AT-hook 1 (HMGA1) is a structural transcription factor that acts as a tumor promotor in a variety of cancers where it is overexpressed, including hepatocellular [6], [2], pancreatic [7], colorectal [8], and gastric [9]. It has been established that in CCA, HMGA1 is a commonly overexpressed [2, 10] ; however, the regulatory mechanism of HMGA1 and the clinical treatment strategies targeting HMGA1 overexpression remain unclear.

Current evidence suggests that mitogens, cytokines, and differentiation inducers could trigger Cyclin D1 (CCND1) protein expression in cancer cells. Subsequently, CCND1, in complex with CDK4/6, phosphorylates retinoblastoma protein (RB), resulting in the induction of E2F target genes and progression from the G1 to S [11]. Given their well-established biological functions, CDK4/6 inhibitors represent a promising molecular targeted drug in tumors. Palbociclib is a highly selective and orally active CDK4/6 suppressor approved by the FDA for treating ER + breast cancer and is also effective against various cancer [12, 13]. However, dysregulation of the RB pathway, such as CDK4/6-CCND1 amplification, and other mechanisms independent of RB, such as activation of the PI3K/AKT/mTOR pathway, have been reported to result in resistance to CDK4/6 inhibitors and, thus, yield limited overall survival [14, 15]. In recent years, Palbociclib has been evaluated as second-line therapy in treating CCND1 gene amplification SCLC patients (https://clinicaltrials.gov/). Nonetheless, the therapeutic significance of palbociclib or other CDK4/6 inhibitors for iCCA remains poorly defined.

Resistance to CDK4/6 inhibitors may induce the activation of signaling pathways independent of CDK4/6 or increase the CDK4/6 inhibition threshold, leading to sustained tumor [14]. It is well-recognized that the PI3K/AKT/mTOR cascade is a commonly aberrant activation pathway in [16]. The specific activation of the PI3K/AKT/mTOR pathway is associated with resistance to CDK4/6 [17, 18]. In a recent study, a combination of CDK4/6 and mTOR inhibitors treatment showed a synergistic effect against iCCA tumor cell proliferation, but other CDK4/6 functions in transcription or differentiation independent of cell cycle processes reported recently remain [5, 19, 20]. PF-04691502 (a PI3K/mTOR inhibitor) is currently under evaluation in several phases I clinical trials of patients with solid tumors as a single agent or in combination therapy (https://clinicaltrials.gov/).

In a recent study, we evaluated the expression and clinical significance of HMGA1 in a large cohort of iCCA [10]. Herein, we examined the oncogenic functions of HMGA1. Experimental and in silico analyses identified CCND1 as the key protein in HMGA1-induced iCCA biological functions through the PI3K/Akt pathway. Given this background, we sought to identify whether palbociclib administration yields antiproliferative and other related biological activities on iCCA cells in vitro and in vivo. In addition, we investigated whether palbociclib works synergistically with the PI3K/mTOR inhibitor PF-04691502 to induce iCCA regression. Last but not least, we substantiated the significant efficacy of combined CDK4/6 and PI3K/mTOR inhibition in vitro and in vivo, suggesting that combining FDA-approved agents may improve the treatment of this lethal malignancy.

## Materials and methods

### Clinical tissue samples

Eighteen human iCCA tissue samples were obtained from The Second Hospital of Shandong University from September 2019 to September 2022. All patient materials used for research purposes were obtained with prior consent from patients. The study was approved by the Ethics Committee of The Second Hospital of Shandong University.

### Cells and agents

Human Intrahepatic biliary epithelial cells (HIBEpiC), RBE cells and Human intrahepatic cholangiocarcinoma cell lines (HCCC-9810) were cultured in RPMI-1640 medium (Thermo Fisher Scientific, Waltham, MA, USA) supplemented with 10% fetal bovine serum(FBS, Thermo Fisher Scientific) and 1% penicillin/streptomycin(Hyclone, SV30010). QBC-939, HUCCT1, HepG2, Hep-3B, PLC/PRF/5 and Huh7 were cultured in DMEM medium (Thermo Fisher Scientific) containing 10% FBS and 1% penicillin/streptomycin. Information on reagents and antibodies is detailed in Supplementary Table S1.

All cell lines were cultured at 37 °C with a humidified 5% CO2 atmosphere. Moreover, the identities of all cell lines were confirmed by short tandem repeat (STR) analysis, and detection of mycoplasma contamination was conducted for cell lines prior to any experiments performed.

### RNA extraction and quantitative PCR(qPCR)

Total RNA was extracted from fresh clinical tissues or cells using TRIzol reagent (Thermo Fisher), and cDNA synthesis was performed using a reverse transcriptase kit (TOYOBO, Japan). Quantitative PCR (qPCR) was performed as previously described. The primers used in qPCR were either designed by PrimerBlast (https://www.ncbi.nlm.nih.gov/tools/primer-blast/) or obtained from published sequences provided in Supplementary Table S2.

### Western blot and analysis

Total proteins were extracted from tissues or cultured cells using RIPA buffer supplemented with 1% PMSF (Beyotime, Shanghai, China) and 1% phosphatase inhibitor (Solarbio) and were subjected to western blot as previously [2].

### Transfection and stable cell lines

HMGA1 short-hairpin RNAs (shRNAs) were constructed using the lentivirus vector LV-5 (GenePharma). The lentiviral pLKO vector containing shRNA could target HMGA1. Stable cell lines were generated as previously [2]. The related sequences are listed in Supplementary Table S3.

### Cell proliferation and colony formation assays

Cell Counting Kit 8 (CCK-8; Dojindo, Japan) assays were performed to assess cell proliferation according to the manufacturer’s instructions. Briefly, 3000–5000 cells were plated into 96-well plates for shRNA treatment and half-maximal inhibitory concentration (IC50) experiments. Cells were treated with reagents in different concentrations and counted after 4 days or treated with shRNA, vehicle (DMSO) or different reagents and measured after 1–5 days.

For the colony formation assay, 1–2 × 10^4^ cells were seeded in 12-well plates and adhered overnight. After 9 days, stable cells or cells treated with DMSO or inhibitors were fixed with polyformaldehyde (1%), washed twice with PBS and stained with crystal violet solution (0.5%) for 15 min at room temperature.

### Cell migration and invasion assays

5–10 × 10^4^ cells were suspended in 200 µL of 3% serum medium and seeded into the upper chambers with or without matrigel coated. The bottom chambers were filled with 600 µL complete medium. After 24–36 h, the cells attached to the bottom of the chambers were fixed with methanol for 30 min and stained with 0.1% crystal violet for 1 h. The cells were photographed and quantified by counting the cell number in five random visual fields.

### Soft agar colony forming assay

Soft agar colony formation assays were used for cell three-dimensional (3D) sphere culture as previously [2, 21]. In addition, different reagents (DMSO or inhibitors) were added to the top layer of low melting point agarose for drug experiments. All experiments were performed in triplicates independently.

### Flow cytometry analysis

Cells were harvested after treatment with DMSO or inhibitors for 3 days.

For cell cycle analysis, cells were fixed with 70% cold ethanol at 4 °C for at least 30 min or overnight and then washed with PBS and stained with RNase/PI Staining Solution for 30 min at room temperature. DNA content of at least 10,000 cells in each sample was measured by flow cytometry (Beckman Coulter) and analyzed using ModFIT LT software.

Cells were incubated with 10 µM EdU for 2 h and then stained with the Click-iT® EdU Imaging Kit (C0075S, BeyoClick) reaction cocktail for 15 min according to the manufacturer’s instructions. All samples were detected on a flow cytometer (Beckman Coulter). Data were analyzed using FlowJo-V10 software.

## Immunofluorescence staining (IF)

For IF, cells were seeded on coverslips in a 24-well plate and cultured with drugs or DMSO for 3 days. Then, cells were washed with PBS, fixed with 4% paraformaldehyde, permeabilized by 0.5% Triton X-100, incubated with 5% goat serum and incubated by primary antibody (2.5% serum dilution). After incubation overnight, cells were incubated with secondary antibodies goat anti-rabbit Alexa Fluor 594 and anti-mouse Alexa Fluor 488 and stained with DAPI. Images were taken with laser scanning confocal microscopy (LSM 800).

### Dual-luciferase reporter assay

5 × 10^4^ cells were seeded in 24-well plates in triplicates and incubated for 24 h. Cells were transiently transfected with the indicated plasmids and the pRL-TK Renilla luciferase plasmid for forty-eight hours and were harvested and processed using a Dual-Luciferase Reporter Assay Kit (Promega, Madison, WI, USA) according to the manufacturer’s instructions as previously [2]. The promoter region sequences are provided in Supplementary Table 4.

### Xenograft models

Female BALB/c nude mice (5 weeks of age) were purchased from Vital River Laboratory Animal Technology Company(Beijing, China). iCCA cells were subcutaneously injected into the right flank of nude mice (n = 6/group). Mice were examined every 3 days for tumor growth, as previously [2]. Tumor diameters were measured with an external caliper, and the tumor volume was calculated using the following formula: (Length × Width^2^) /2. Mice were randomly separated into groups when their tumor volume reached approximately 150mm^3^ before drug administration.

For in vivo hepatic metastasis assays, 5–10 × 10^5^ iCCA cells were injected into the caudal vein of nude mice (n = 6/group). Tumor metastasis was finally confirmed with HE staining based on nuclear atypia during histopathological analysis. Subsequently, drugs were administered daily by vehicle (0.5% CMC-Na and 0.2% Tween 80, oral gavage), palbociclib (40 mg/kg, oral gavage), PF-04691502 (20 mg/kg, oral gavage), or combinations of palbociclib and PF-04691502 (n = 6 per group). Mice were treated until the tumor reached the endpoint (for metastasis assays) and were sacrificed for tissue harvesting. Dissected tumors/organs were stored in liquid nitrogen or fixed in 10% buffered formalin for routine histopathological processing.

All nude mice were maintained under specific pathogen-free conditions at the Experimental Animal Department of The Second Hospital of Shandong University. All animal experiments were approved by the Clinical Research Ethics Committee of The Second Hospital of Shandong University.

### Statistical analysis

Statistical analysis was performed using SPSS 17.0 and GraphPad Prism 5/8 software (GraphPad Prism Software, San Diego, CA, USA). Data were represented as mean ± SD or SEM as indicated in the figure legends. ImageJ software (National Institutes of Health, Bethesda, MD) was used to quantify the western blot films.

## Results

### HMGA1 is critical for the progression of iCCA

**Fig. 1:**
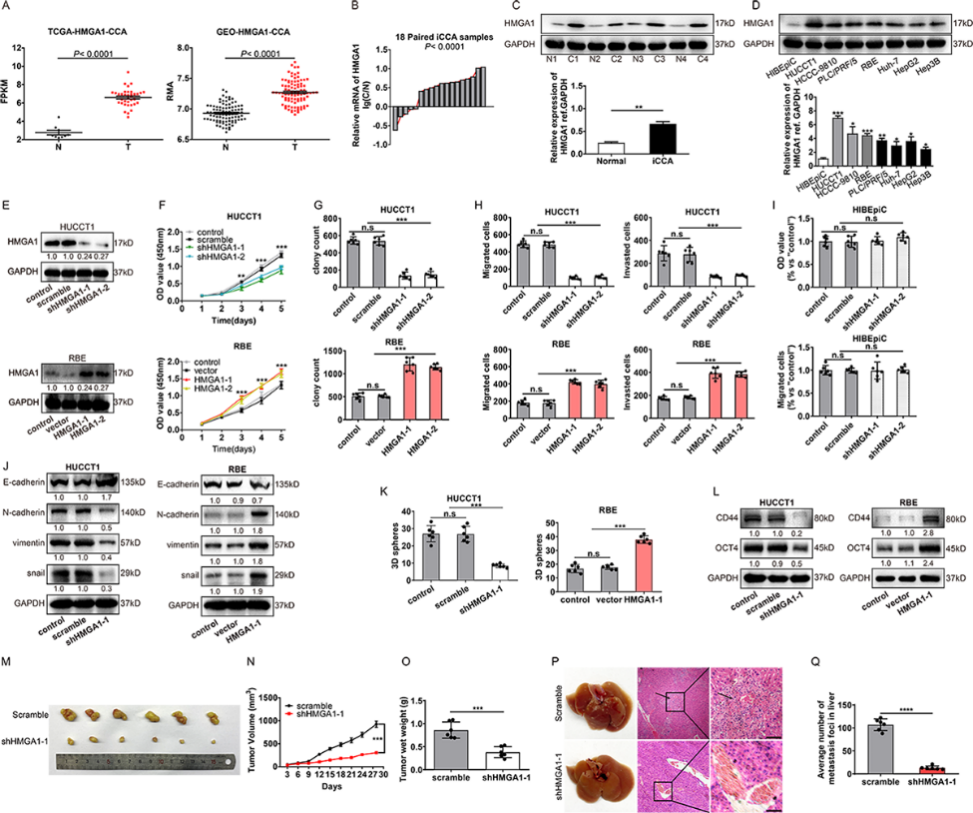
**HMGA1 is a potential activator of iCCA progression** A, Amplification of HMGA1 is common in TCGA and GEO provisional iCCA cohort B-C, The expression of HMGA1 was detected with qPCR in 18 pairs of iCCAs and adjacent tissues and with WB in four randomly-selected pairs of iCCA tissues D, HMGA1 expressions in intrahepatic bile duct cell line HiBEpiC and different hepatobiliary tumor cell lines: HUCCT1, HCCC-9810 and RBE iCCA cell lines and PLC/PRF/5, Huh7, HepG2 and Hep3B.hepatocellular carcinoma cell lines E, Western blot showed that HMGA1 expression was silenced in HUCCT1 cells or overexpressed in RBE cells F-I, HMGA1 enhanced the proliferation, migration and invasion of iCCA cell lines. Cell proliferation was evaluated with CCK-8 assay and colony formation assay. Cell migration and invasion were investigated with transwell assays J, Effects of HMGA1 on EMT were detected by western blot after silencing or overexpressing HMGA1 in iCCA cells K-L, 3D sphere formation and western blot indicated HMGA1 increased the stemness of iCCA. M-Q, Xenografts and metastasis models were established with stable HMGA1-silenced HUCCT1 cells. Mice engrafted with HMGA1-silencing cells exhibited lower tumor volumes, tumor weights and metastatic lesions Data were shown as mean ± SEM. n.s. represents not significant. *p < 0.05, **p < 0.01, ***p < 0.001 and ****p < 0.0001 as calculated by the one-way or two-way ANOVA. Scale bar: 50 μm

To investigate the function of HMGA1 in iCCA progression, we first analyzed the copy number alterations of HMGA1 in The Cancer Genome Atlas (TCGA) Research Network (http://cancergenome.nih.gov) and Gene Expression Omnibus datasets (GSE76311). Analysis of these public datasets showed that HMGA1 is highly expressed in iCCA, suggesting that HMGA1 may play a role in tumor progression (Fig. 1A). We further detected the expression of HMGA1 in iCCAs and paired tumor-adjacent tissues with qPCR and western blot (WB) which demonstrated that HMGA1 was significantly upregulated in iCCAs (Fig. 1B, C). In a previous study, we documented that HMGA1 amplification and gain were associated with unfavorable prognosis, suggesting that HMGA1 may be a prognostic biomarker of [10]. In the present study, WB analysis showed that HMGA1 was expressed ubiquitously at different levels in hepatobiliary cancer cell lines and was significantly higher than in the HIBEpiC cell line(Fig. 1D). Both HUCCT1 and RBE cell lines exhibited HMGA1 expression using the DepMap web resources (Supplementary Fig. 1A)(https://depmap.org). In these two cell lines, HMGA1 was silenced with shRNA or overexpressed with a lentivirus carrying the HMGA1 cDNA respectively (Fig. 1E). Cell Counting Kit-8 (CCK-8), colony formation and transwell assays demonstrated that HMGA1 knockdown suppressed iCCA proliferation, migration and invasion, whereas HMGA1 overexpression yielded the opposite effects (Fig. 1F-H, Supplementary Fig. 1B, C). Knockdown of HMGA1 did not significantly inhibit the proliferation and motility in the intrahepatic biliary epithelial cells (Fig. 1I, Supplementary Fig. 1D). We further investigated the effect of HMGA1 on the EMT and stemness of iCCA cells (Fig. 1J-L, Supplementary Fig. 1E), given that our previous study showed that HMGA1 is an important factor for the stemness and EMT of pCCA [2]. Stable HMGA1 silencing HUCCT1 cells were injected for in vivo subcutaneous xenografts and tail vein metastasis (Fig. 1M-Q). The xenografts with HMGA1-silencing HUCCT1 cells exhibited decreased tumor volumes, weights and metastatic lesions compared with shRNA, substantiating the procarcinogenic role of HMGA1 in iCCA. The above results indicated that HMGA1 could significantly influence the stemness, EMT, proliferation, migration and invasion of iCCA.

### HMGA1 promoted the transcription and expression of CCND1

**Fig. 2:**
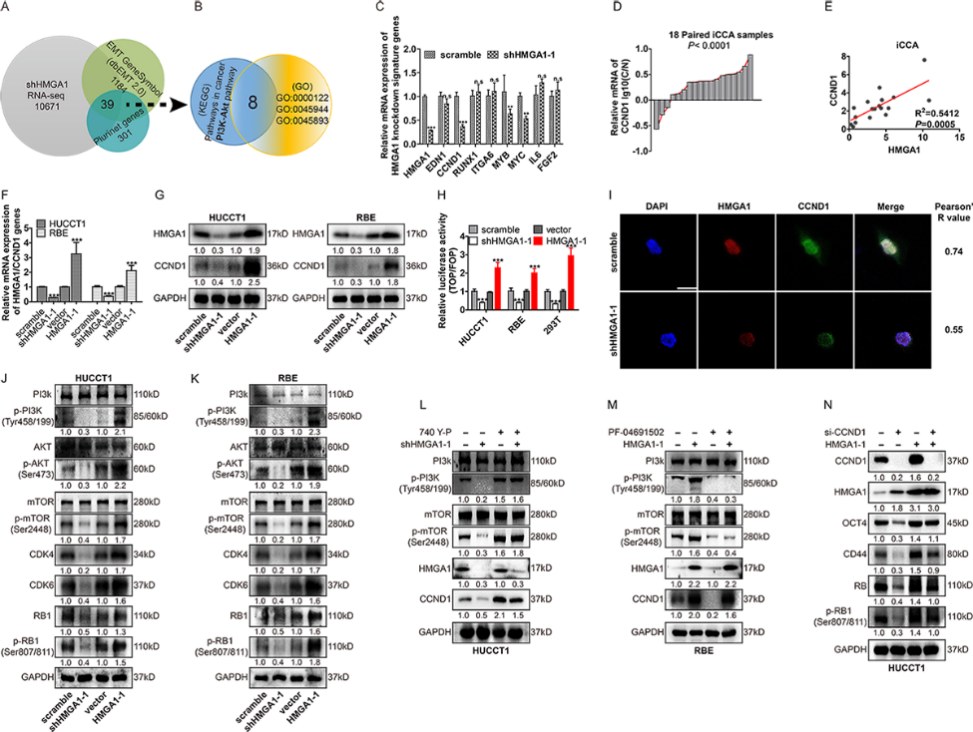
**HMGA1 promoted the transcription and expression of CCND1.** A, 39 genes were filtered out among the datasets of shHMGA, EMT and PluriNet signature genes. B-C, 8 genes were screened from the top lists of the KEGG and the GO enrichment analysis and evaluated in RBE. D, CCND1 mRNA levels in 18 pairs of iCCAs and adjacent normal bile duct tissues were detected with qRT-PCR and showed as log (CCND1 Tumor/CCND1Non-tumor). E, Correlation between HMGA1 and CCND1 mRNA ratio in fresh iCCA tissues calculated by mRNA (tumor)/mRNA (adjacent tissue). F-G, qRT-PCR and WB showed that HMGA1 expression regulated the expression of CCND1 in HUCCT1 and RBE cells. H, HMGA1 promoted the transcription of CCND1 in both HUCCT1 and RBE cells and 293 T cells. The transcriptional activity of CCND1 was detected with luciferase assays. I, The expression and localization of HMGA1 and CCND1 in HUCCT1 cells were detected with immunofluorescence. The co-localization of HMGA1 and CCND1 was attenuated after HMGA1 knockdown. Scale bar 20 μm. J-K, WB showed that the PI3K signal pathway was regulated by HMGA1. L-N, HMGA1-silenced or HMGA1-overexpressing HUCCT1 or RBE cells were incubated in PI3K agonist 740 Y-P (30 µM, 24 h) or PI3K inhibitor PF-04691502 (0.5 µM, 48 h), and CCND1 expression was detected with WB. In HMGA1-overexpressing HUCCT1 cells, CCND1 was knocked down, and OCT4, CD44 and (p)RB expression were detected with WB. n.s. ,** and *** represented not signifificant, P < 0.01 and < 0.001, respectively. Analyzed data were from three independent experiments and shown as means ± SEM. Data were from three independent experiments and analyzed with the T-test(C, F-K). Spearman correlation analysis was performed in (E and I)

In a previous study, we performed mRNA sequencing of HMGA1-knockdown in HUCCT1 cells. 39 genes were screened out among the datasets of shHMGA1, EMT and PluriNet signature genes (Fig. 2A). Next, we identified significantly enriched KEGG (Pathways in cancer and PI3K/Akt signaling pathway) and GO (GO:0000122, GO:0045944 and GO:0045893) terms, and identified eight genes (EDN1, CCND1, MYB, MYC, RUNX1, ITGA6, IL6 and FGF2 (Fig. 2B, Supplementary Fig. 2A, B). Then, the above 8 genes were further evaluated in RBE cells. Interestingly, CCND1, MYB and MYC showed a positive correlation with HMGA1 in RBE cells (Fig. 2C). Although these genes exhibited different degrees of upregulation in iCCA, analysis in TCGA and GEO datasets showed that only CCND1 was significantly associated with HMGA1 in iCCAs (Supplementary Fig. 2C-F). Moreover, qRT-PCR showed that CCND1 expression was upregulated in iCCA tissues(Fig. 2D).

A previous study showed that HMGA1 could activate the transcription of the cyclin D1 gene by directly binding to its [22]. Our results substantiated a strong positive correlation between HMGA1 and CCND1 expression in iCCA tissues (Fig. 2E) and CCND1 expression was regulated by HMGA1 (Fig. 2F, G). Luciferase and immunofluorescence assays highlighted that HMGA1 promoted CCND1 expression in iCCA cells (Fig. 2H, I). The western blot analysis showed that HMGA1 altered the phosphorylation of PI3K, Akt and mTOR and the expression of CCDN1, CDK4 and CDK6(Fig. 2J, K). To further validate the role of PI3K/Akt signaling pathway in HMGA1-mediated CCND1 expression, we silenced HMGA1 expression or/and stimulated PI3K/Akt activation in HUCCT1 cells. Both HMGA1 knockdown or PI3K/Akt activity activation altered the expression of CCND (Fig. 2L). In contrast, HMGA1-overexpressing RBE cells were treated with the PI3K/Akt signaling antagonist PF-04691502 (Fig. 2M). The results showed that CCND1 expression was induced by HMGA1 through the PI3K/Akt signaling pathway identified by KEGG analysis in iCCA cells.(Fig. 2J-N). The above results suggested that HMGA1 induced CCND1 expression by promoting its transcription and activating the PI3K signaling pathway.

### CDK4/6 inhibitors as a putative target for iCCA

A previous study indicated that activating the CDK4/6 pathway is ubiquitous in human iCCA, suggesting it is a potential target in this lethal malignancy. First, we showed that the CCND1 was expressed ubiquitously in hepatobiliary cancer cell lines using western blot.

Next, the CRISPR dataset (https://depmap.org) was used to investigate the target gene CCND1. We found that the gene effect of CCND1 was significant in CCA (Supplementary Fig. 3A). We then used the CRISPR data across hepatobiliary cancer models to explore the relationship among CCND1, CDK4 and CDK6 and found a significant correlation between CCND1 and CDK4 /CDK6 dependence (R2 = 0.1471, p = 0.0055; R2 = 0.2188, p = 0.0002)(Supplementary Fig. 3B, C). To assess the relationship between CCND1 and CDK4/6 inhibitor sensitivity, we compared pharmacological data to CRISPR screening data and found that CCND1 correlated with palbociclib, a CDK4/6 inhibitor, and determined sensitivity in hepatobiliary cancers (Supplementary Fig. 3D).

**Fig. 3:**
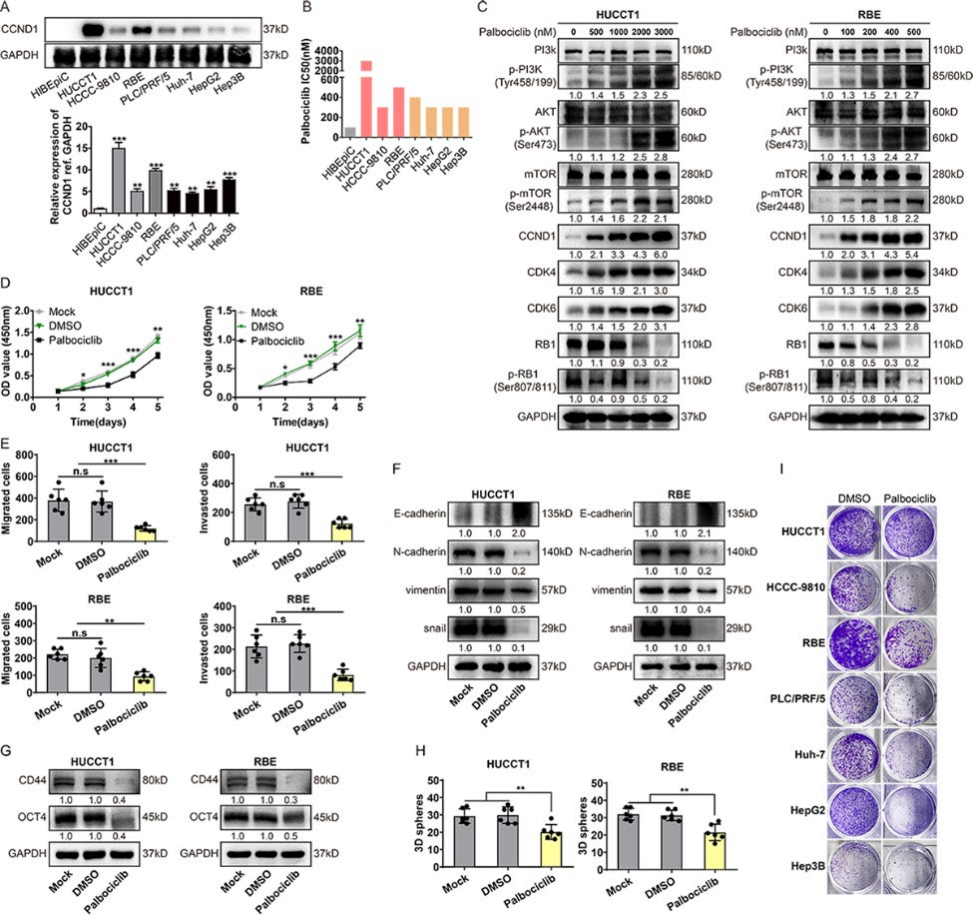
**CDK4/6 inhibitors as a putative target for intrahepatic cholangiocarcinoma (iCCA). ** A, Representative western blot analysis of Cyclin D1 in hepatobiliary cancer cell lines. B, Palbociclib drug sensitivity half maximal inhibitory concentration (IC50) values (nM). C, Western blot analysis of genes involved in the PI3K/Akt pathway and cell cycle-related proteins in iCCA cell lines HUCCT1 and RBE cells treated with palbociclib or with water control. Protein lysates were collected after drug treatment for 48 h. D-E, palbociclib inhibited the proliferation, migration and invasion of iCCA cell lines. Cell proliferation was evaluated with CCK − 8 assay. Cell migration and invasion were investigated with transwell assays. F-G, Western blot analysis of EMT-related factors and cell stemness-related proteins in iCCA cell lines HUCCT1 and RBE cells treated with palbociclib (3000 nM/500 nM), or control. Protein lysates were collected after drug treatment for 48 h. H-I, Images showing 3D sphere formation and colony formation assays of iCCA cell lines, after 3000nM (HUCCT1) or 500 nM (RBE) palbociclib treatment for 9 days. Data were shown as mean ± SEM. n.s. represents not significant. *p < 0.05, **p < 0.01 and ***p < 0.001 as calculated by the one-way or two-way ANOVA. Data from one representative experiment are presented (n = 3)

We next evaluated the impact of palbociclib across a panel of hepatobiliary cancer cell lines. As shown in Fig. 3B, hepatobiliary cancer cell lines showed different responses to palbociclib, with an IC50 ranging from 100 nM to 3 μm. We then investigated the signaling changes in cell line models with palbociclib. As shown in Fig. 3C, although 2000 nM (HUCCT1) and 400 nM (RBE) palbociclib could attenuate total retinoblastoma protein and phosphorylated retinoblastoma protein (pRB), 3000 nM (HUCCT1) and 500 nM (RBE) palbociclib could potently block pRB and total RB, respectively. Moreover, palbociclib activated the phosphorylation of PI3K, Akt and mTOR and upregulated the expression of its target proteins CCDN1, CDK4 and CDK6. Cyclin D-CDK4/6 complexes have been reported to directly interact with various transcription [23], regulating the transcription of other genes in a kinase-independent manner and participating in other tumor cell functions, including differentiation, DNA damage repair and control of cell [20]. To investigate the effects of palbociclib on other cellular functions that might be relevant to cancer therapy, we performed follow-up experiments for validation. After HUCCT1 and RBE cells were treated with palbociclib, the CCK-8 and transwell assays showed that palbociclib could suppress iCCA proliferation, migration and invasion, especially during the first 3 days (Fig. 3D-E, Supplementary Fig. 4A, B). Moreover, expression of EMT-related factors and cell stemness-related proteins, such as OCT4 and CD44, was reduced (except E-cadherin was increased) after treatment with palbociclib (Fig. 3F-G), consistent with the CRISPR dataset (Supplementary Fig. 3E, F). We further explored the long-term efficacy of palbociclib monotherapy in hepatobiliary cancer cell lines in vitro. Unlike the CCK8 assay results (Fig. 3D), we evaluated growth after 9 days of treatment with 3000nM (HUCCT1) or 500 nM (RBE) palbociclib (with culture medium and drugs renewed every 3 days)(Fig. 3H-I, Supplementary Fig. 4C). Interestingly, although there was more stable attenuation of growth in the HIBEpic model, we observed substantial outgrowth in each hepatobiliary cancer cell model. Indeed, these data indicate that CDK4/6 inhibition represents a putative target of iCCA and highlights that combination therapy has huge prospects.

### Potential role of PI3K/mTOR inhibitors in iCCA treatment

We next sought to identify essential targets/genes within iCCA for combination treatment. Early studies identified CCND1/CDK4/CDK6 as a critical activity complex for entry into the cell cycle downstream of mammalian cells signaling pathways such as PI3K/[14]. Interestingly, acquired resistance of CDK4/6 inhibitors is widely thought to function via activation of the upstream PI3K/AKT [24], which could be resensitized by targeting activated PI3K/mTOR signaling in CCA cells resistant to CDK4/6 [25]. As shown in our results (Supplementary Fig. 3G), for CCND1/CDK4/CDK6, higher mRNA expression was associated with greater PF-04691502 (PI3K/mTOR inhibitor) sensitivity. Consistently, when our analysis was expanded to all solid tumor cells, we observed a significant positive correlation between the sensitivity to palbociclib and PF-04691502 (Supplementary Fig. 3H).

**Fig. 4:**
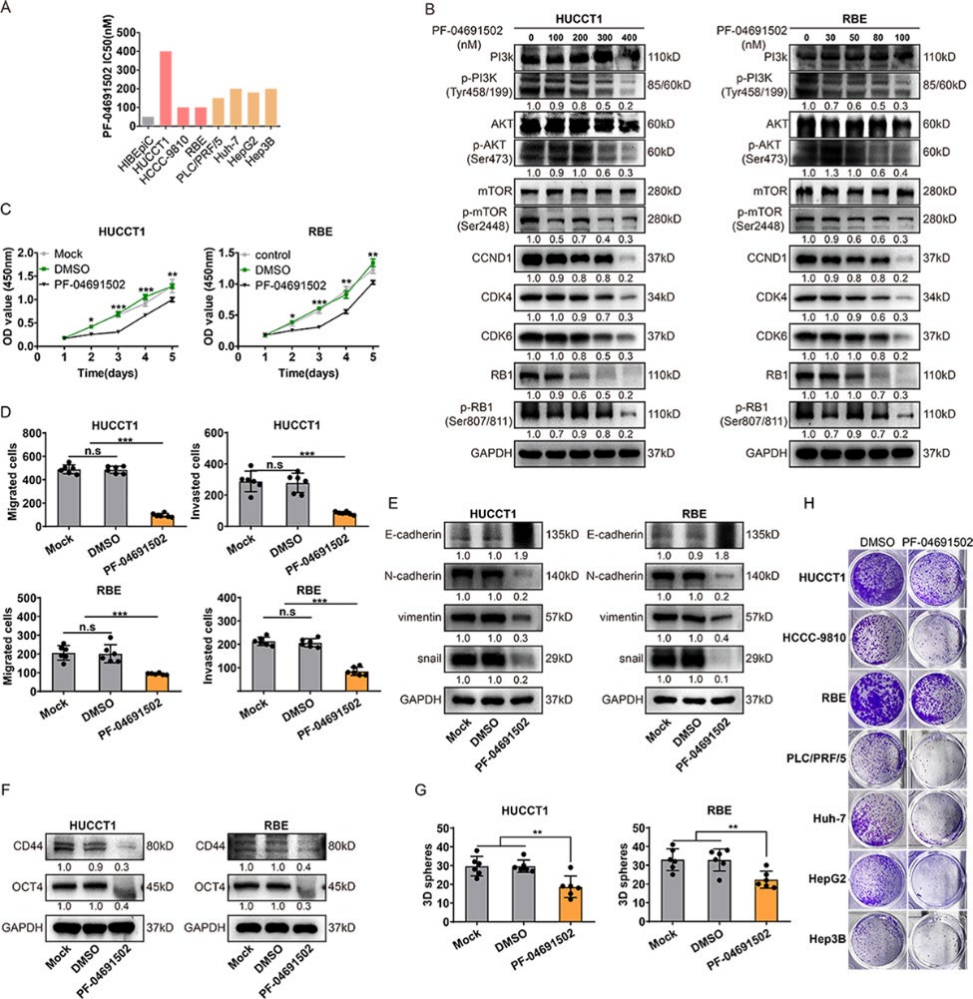
**Potential role of PI3K/mTOR inhibitors in iCCA treatment. ** A, PF-04691502 drug sensitivity half maximal inhibitory concentration (IC50) values (nM). B, Western blot analysis of related genes in iCCA cell lines treated with PF-04691502. Protein lysates were collected after drug treatment for 48 h. C-D, PF-04691502 inhibited the proliferation, migration and invasion of iCCA cell lines. Cell proliferation was evaluated with CCK − 8 assay. Cell migration and invasion were investigated with transwell assays. E-F, Western blot analysis of EMT-related factors and cell stemness-related proteins in iCCA cell lines HUCCT1 and RBE cells treated with PF-04691502, or control. Protein lysates were collected after drug treatment for 48 h. G-H, Images showing 3D sphere formation and colony formation assays of iCCA cell lines, after 3000nM(HUCCT1) or 500 nM(RBE) PF-04691502 treatment for 9 days. Data were shown as mean ± SEM. n.s. represents not significant. *p < 0.05, **p < 0.01 and ***p < 0.001 as calculated by the one-way or two-way ANOVA. Data from one representative experiment are presented (n = 3)

To further investigate the effect of the PI3K/AKT pathway on iCCA, we first compared the IC50 of PF-04691502 in hepatobiliary cancer cell lines (Fig. 4A). We examined the relevant protein levels in iCCA cells treated with different concentrations of PF-04691502 to investigate the signaling changes in cell line models. PF-04691502 decreased the phosphorylation of PI3K, Akt, and mTOR and downregulated the expression of CCND1, CDK4, and CDK6, opposite to findings observed with palbociclib treatment (Fig. 4B). We next assessed whether PF-04691502 was a putative target for iCCA. Interestingly, in subsequent proliferation, migration, invasion, colony formation and 3D sphere formation assays, PF-04691502 exhibited similar effects to palbociclib (Fig. 4C-H, Supplementary Fig. 4D-F), especially in the long-term efficacy of inhibitor monotherapy in hepatobiliary cancer cell lines in vitro (Fig. 4G, H, Supplementary Fig. 4F).

### Dual inhibition of CDK4/6 and PI3K/AKT pathways yields efficacy in iCCA

**Fig. 5:**
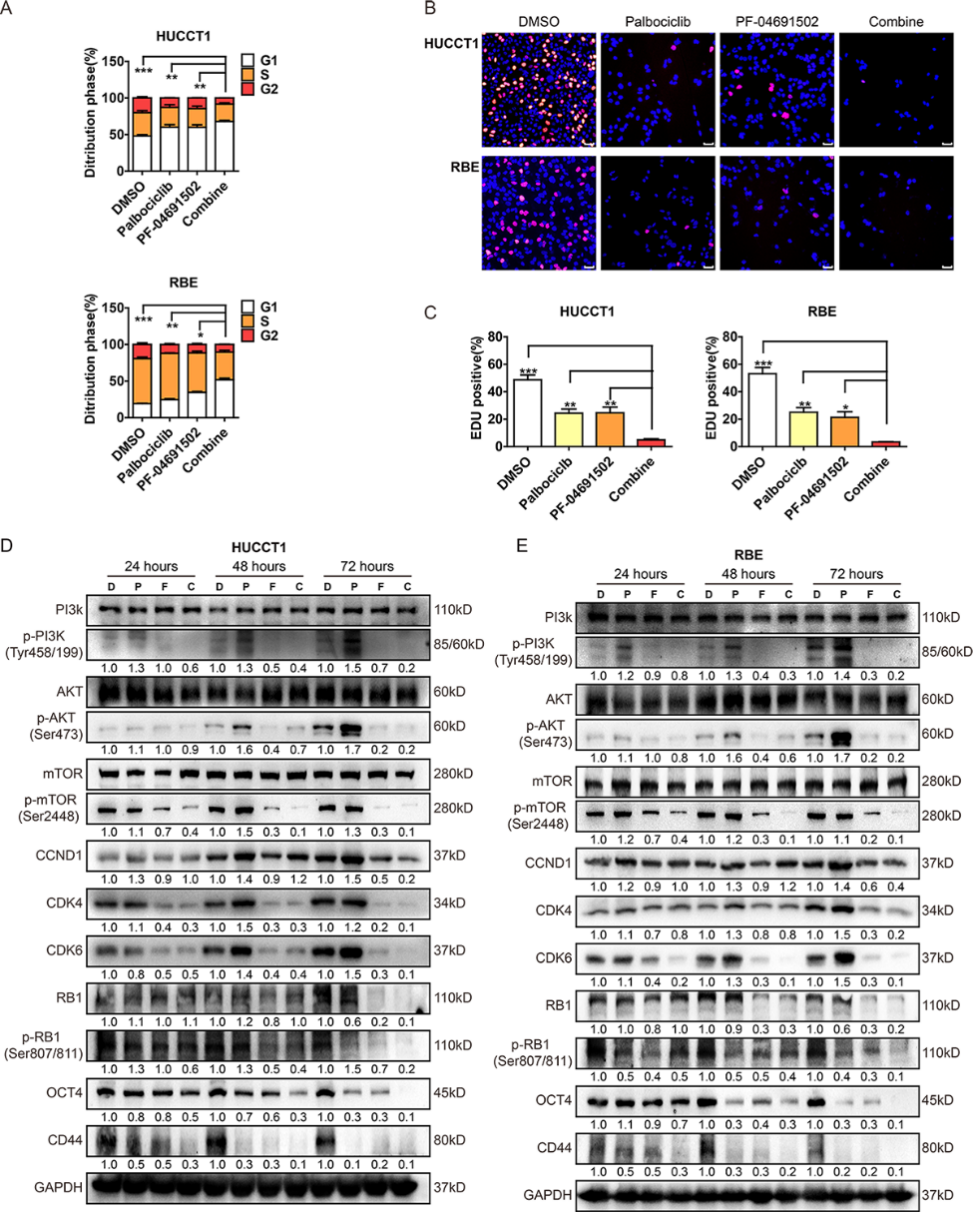
**PI3K/AKT and CDK4/6 pathway dual inhibition demonstrate efficacy in iCCA. ** A, Representative histograms depicting cell cycle profiles of iCCA cell lines on treatment with palbociclib (3000 nM), PF-04691502 (500 nM), the combination or with DMSO control for 48 h. B-C, Immunofluorescence staining assay of iCCA cells treated with a single agent (PF-04691502 or palbociclib) or a combination of both compounds at a fixed ratio (1:1). D-E, Western blot analysis of genes involved in PI3K/Akt pathway, cell cycle-related proteins and stemness biomarkers in iCCA cell lines treated with a single agent (PF-04691502 or palbociclib) or a combination of both compounds at a fixed ratio (1:1). *p < 0.05; **p < 0.01; ***p < 0.001; Scale bar: 200 μm

Based on the results above, we speculated that in iCCA cell lines, the combination of PF-04691502 and palbociclib achieved significantly higher cytostatic effects than either drug alone across a wide range of doses. We next evaluated the cell cycle distribution under single or combination drug treatment. The PF-04691502 and palbociclib combination treatment significantly induced G1/S cell cycle arrest and limited cell proliferation compared with monotherapy in iCCA cell lines following 48 h of treatment, suggesting that the combination of PF-04691502 and palbociclib inhibited cell proliferation by inducing cell cycle arrest (Fig. 5A-C, Supplementary Fig. 5A). We next performed biochemical studies to confirm target engagement, and found that PF-04691502 reduced activation of the PI3K pathway, as measured by phosphorylation of PI3K, AKT and mTOR. Besides, the addition of palbociclib significantly impacted the activity of PI3K markers, while PF-04691502 augmented the effects of palbociclib on the inhibition of total RB and p-Rb. We also observed that palbociclib increased the CDK4, CDK6 and cyclin D1 protein levels and decreased CD44 and OCT4, explaining the ability of CDK4/6 treatment to block RB phosphorylation and stemness induced by blocking PI3K signaling (Fig. 5D, E).

**Fig. 6:**
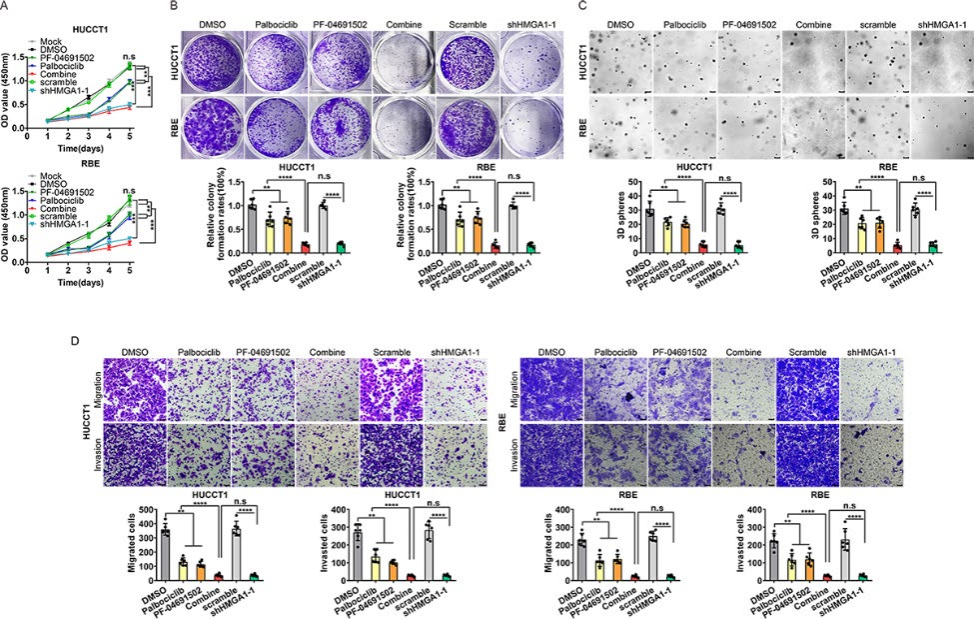
**PF-04691502 works synergistically with palbociclib to inhibit iCCA growth, EMT and stemness in vitro. ** CCK − 8 assay (A), colony formation assay(B), 3D sphere formation assay(C), and transwell assay(D) analysis of iCCA cells treated with a single agent (PF-04691502 or palbociclib), a combination of both compounds at a fixed ratio (1:1) or shRNA-induced silencing of HMGA1. Analyzed data were from three independent experiments and shown as means ± SEM. Analysis for statistical significance was performed using Student’s t-test (n.s.,**, *** and **** represented not significant, P < 0.01, < 0.001and < 0.0001, respectively )

Given the ability of PF-04691502 to reverse palbociclib resistance, we evaluated the effects of combination treatment with PF-04691502 and palbociclib in iCCA cells. To determine which phenotypes the combination of the two drugs might affect, we examined proliferation, migration, invasion, EMT effect and stemness under single or combination drug treatment. As shown in Fig. 6A, the combination of PF-04691502 and palbociclib yielded a more significant effect on iCCAs than any single regimen or drug after 5 days. Furthermore, compared with either inhibitor alone, the combination of PF-04691502 and palbociclib at low concentrations significantly attenuated clonogenicity (Fig. 6B), consistent with the results obtained for the 3D sphere formation. The 3D culture showed that the sphere-formation efficiency of the combination groups was severely decreased compared to any single inhibitor treatment group in iCCA cells (Fig. 6C). Consistently, co-treatment with PF-04691502 and palbociclib resulted in more significant suppression of migration and invasion than either mono-drug treatment (Fig. 6D). Meanwhile, the results showed that knockdown of HMGA1 had yielded the same effect on iCCA proliferation, migration and invasion to, compared with the combination of palbociclib and PF-04691502 (Fig. 6A-D).

### PF-04691502 and palbociclib show synergistic effects in vivo

**Fig. 7:**
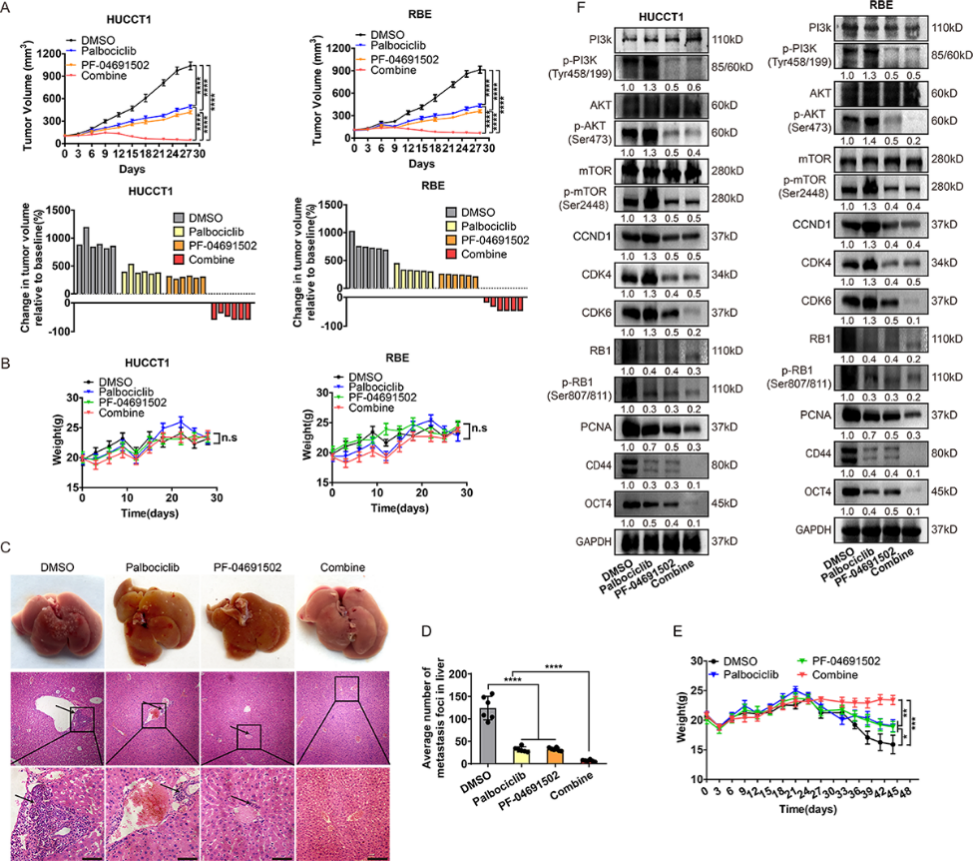
**PF-04691502 and palbociclib show synergistic effects in vivo. ** A, Top: growth curve for subcutaneous xenograft tumors treated with vehicle, palbociclib (50 mg/kg), PF-04691502 (20 mg/kg) or the combination. Bottom: waterfall plot showing the tumor volume change (at day 35) relative to baseline volume (at day 7). Each bar represents one xenograft tumor. B, Changes in body weight of indicated groups of nude mice models with subcutaneous xenograft tumors were observed. C, Representative HE staining images of liver samples from each group. D, Numbers of metastatic lesions on the surface of the liver. E, Changes in body weight of nude mice model with tail vein injection were shown. F, Representative indicated proteins expression of tumors assessed via western blot. Data were shown as mean ± SEM. n.s. represents not significant. *p < 0.05, **p < 0.01, ***p < 0.001 and ****p < 0.0001 as calculated by the two-way ANOVA test. Scale bar: 50 μm

To further evaluate the effects of PI3K and CDK4/6 pathway blockade, we evaluated the effect of monotherapy or combination therapies in vivo in nude mice harboring xenografts of iCCA cell lines. Although monotherapy with palbociclib (50 mg/kg) or PF-04691502 (20 mg/kg) delayed tumor growth at 3–4 weeks, progression was still observed. Besides, the combination group yielded a consistent reduction in tumor volume and exhibited more significant tumor growth inhibition than any single regimen or drug (Fig. 7A, Supplementary Fig. 5B). Meanwhile, no significant changes in the body weight of the nude mice were observed, indicating the overall safety of the administered dose (Fig. 7B). Then, we evaluated the metastatic liver lesions of the nude mice model by HE staining. As shown in Fig. 7C-E, the number of metastatic lesions in the combination group was significantly less than in the monotherapy or control groups, resulting in a more stable body weight. To further evaluate the mechanisms underlying the efficacy of combination therapy, we performed western blot of subcutaneous xenograft tumor tissues, dissected from the nude mice following single agent or combination therapies in vivo. As expected, combination therapy yielded a better inhibitory effect on the levels of RB, p-RB and PCNA in xenografts (Fig. 7F), indicating that the combination group could inhibit proliferation resulting in a potentially lethal effect in vivo. Meanwhile, the dual effects of palbociclib and PF-04691502 decreased cell cycle and stemness-related protein expression in vivo, accounting for the ability of palbociclib and PF-04691502 to inhibit proliferation and stemness (Fig. 7F).

**Fig. 8:**
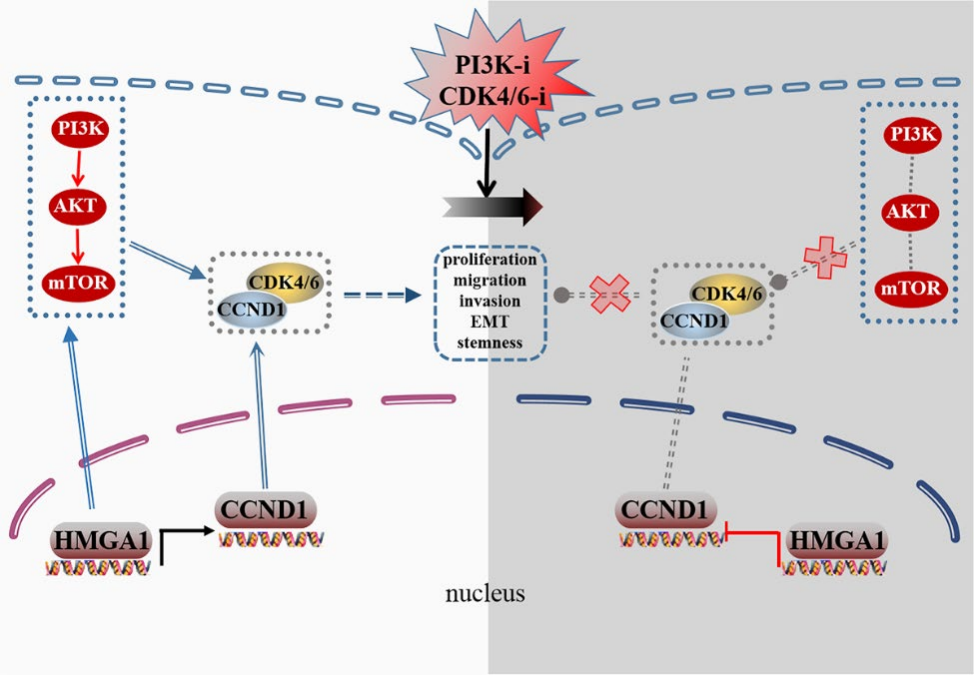
**Schematic depiction of the mechanism by which HMGA1 induces tumor progression mediated by the CCND1/CDK4/CDK6 pathway. ** HMGA1 promotes iCCA progression via promoting CCND1 transcription and activating the PI3K signaling pathway. HMGA1 can induce the transcription and expression of CCND1 and eventually promote iCCA progression. Besides, CCND1/CDK4/CDK6-elevated expression after CDK4/6 inhibitors is thought to function via activation of the upstream PI3K/AKT pathway. Moreover, CDK4/6 inhibitors exert enhanced therapeutic effects in iCCA when combined with PI3K inhibitors to inhibit tumor progression

## Discussion

Intrahepatic CCA (iCCA), which is anatomically located proximal to the secondary bile duct, constitutes a diverse group of malignancies of the biliary tree. Current evidence suggests that the incidence and mortality rates of iCCA are [26], especially in South Korea, China and [27]. The risk factors associated with the development of iCCA include fibroinflammatory biliary tract disease (such as hepatolithiasis, primary sclerosing cholangitis, and liver fluke infractions), viral hepatitis, diabetes, and obesity-associated liver [28, 29]. Intrahepatic CCA is a rare malignancy that is difficult to diagnose early, accounting for a small proportion of patients indicated for radical [30]. It is well-established that delayed clinical diagnosis of iCCA leads to poor prognosis for patients with a 5-year overall survival rate (OS) of 3%[4].

Despite our understanding of the genomic features of iCCAs by tumor genome analysis, effective targeted therapy options are [30]. Although genomic data suggest the potential to introduce PI3K or CDK4/6 inhibition, single-drug therapy exhibits limited efficacy, highlighting the importance of combination [31, 32]. Herein, we demonstrated the efficacy of the combination of CDK4/6 inhibitors with PI3K inhibitors to treat iCCAs (especially HMGA1 overexpressing tumors) based on data from related research, shRNA profiling of HMGA1 (the oncogene that we have been studying), CRISPR screens and pharmacological dataset.

Importantly, we provided compelling evidence that HMGA1 was upregulated in iCCA tissues and contributed to iCCA progression, consistent with our previous study on [2]. Besides, we found that HMGA1 could induce CCND1 expression by promoting CCND1 transcription and activating the PI3K signaling pathway simultaneously in iCCA. Next, the effect of palbociclib for monotherapy was limited due to acquired resistance in iCCA. Furthermore, increased cell cycle-related proteins and activation of the PI3K pathway in iCCA were associated with acquired palbociclib resistance, which PF-04691502 could reverse. Finally, co-treatment with palbociclib and PF-04691502 could effectively induce cell cycle arrest and inhibit EMT and stemness compared with monotherapy, which has significant value for establishing in vivo models.

In this study, we corroborated that HMGA1 promotes the proliferation, EMT, metastasis and stemness of iCCA. An in-depth analysis of our previous sequencing data on shHMGA1 in iCCA combined with TCGA, GEO data analysis and related experimental verification revealed that HMGA1 expression was positively correlated with CCND1 expression at the mRNA level. Interestingly, we next validated that this coexpression was caused by co-amplification and the ability of HMGA1 to regulate the expression of CCND1 at the mRNA and protein levels. It has been reported that HMGA1 could activate the transcription of the cyclin D1 gene by directly binding to its [22]. Our study demonstrated that HMGA1 induced CCND1 expression via promoting CCND1 transcription and activating the PI3K signaling pathway.

Consistent with the literature, we validated the efficacy of CDK4/6 inhibition in CCA preclinical models, especially by in vitro short-term proliferation [25]. Meanwhile, other functions of CDK4/6 involving the promotion of tumor cell [33, 34], [35, 36] and [37, 38] independent of cell cycle processes were revealed. One study reported that CDKN2A mutation was a potential biomarker for CDK4/6 inhibitor sensitivity in [31]. Our findings revealed a possible correlation between CCND1, CDK4 and CDK6 amplification and palbociclib sensitivity. In addition, palbociclib treatment resulted in the downregulation of total RB and p-RB in a dose-dependent manner in our iCCA cell lines. However, despite these correlations, our in vitro results on long-term growth highlight the limited potential of single agents.

There is a growing consensus that cancer cells harbor unique characteristics that increase their susceptibility to agents with specific biological functions, especially when these targets act synergistically on cells; blockade may augment the efficacy of monotherapy. For example, preclinical studies and clinical trials have confirmed that in OR-positive breast cancer, the addition of CDK4/6 inhibitors to endocrine therapy with an OR blocker that inhibits CCND1 activation yields more significant efficacy than [39, 40]. Recent studies on the genomic characterization of biliary tract cancers have found commonalities of predisposing mutations, such as PI3KCA and PIK3C2G [31, 32]. It is now understood that activation of the PI3K pathway contributes to resistance to CDK4/6 inhibitors, which could be reversed by PI3K/mTOR [18, 41]. Importantly, CDK4/6 inhibitors could exert enhanced therapeutic effects in CCA when used with mTOR inhibitors that inhibit cell [5]. A PDX cancer mouse model study showed that resistance to CDK4/6 inhibitors could be alleviated by adding PI3K inhibitors, regardless of PIK3CA mutation [42]. In terms of mechanism, CDK4/6 and phosphoinositide 3-kinase enhancer (PIKE) could promote tumorigenesis by co-amplification and formation of a protein [43]. Importantly, we found that the PI3K pathway was activated in palbociclib monotherapy in vitro of iCCA. Our data substantiated the participation of CDK4/6 inhibition in iCCA and validated our hypothesis, providing the theoretical basis for the development of combination therapy, for instance, with the PI3K pathway inhibitor PF-04691502.

Herein, we harnessed RNA sequencing technologies, cancer data analysis, analysis of public CRISPR data, pharmacological profiling and relevant experiments to uncover novel treatment strategies related to HMGA1 in iCCA. We provided compelling evidence that PI3K/mTOR inhibitors exhibited significant efficacy across iCCA models. Activation of the PI3K pathway could directly regulate the downstream CCND1/CDK4/6 axis, suggesting that pharmacological blockade of this axis is a promising therapeutic strategy in iCCA. It has been shown that the PI3K/AKT/mTOR cascade is a commonly aberrantly activated pathway in [16]. Anti-PI3K pathway antibodies in combination with [44, 45] or other combination [46] have been evaluated for their efficacy against CCA. PF-04691502 (a PI3K/mTOR inhibitor) is currently under evaluation in several phase I clinical trials of patients with solid tumors as a single agent or in combination therapy (https://clinicaltrials.gov/). Our experiments showed a significant synergism between palbociclib and PF-04691502 treatment. Mechanistically, our results suggest that more significant inhibition of the common downstream signaling pathways is observed with the combination compared to monotherapy. As such, the success of this combination, similar to the combination targeting BRAF and MEK in melanoma and colorectal cancer, may be attributed to the synergistic effects on the same cellular pathway leading to more significant [47, 48].

The most important potential challenge to combination strategy and monotherapy is patient tolerance to these regimens and drug [49]. The efficacy of the current therapeutic approach for iCCA is relatively poor. Pemigatinib is an FGFR2 inhibitor and the only FDA-approved targeted drug for CCA. Nonetheless, little is known about tumor resistance to Pemigatinib. In contrast, the most common adverse events include fatigue and cytopenias for palbociclib, and gastrointestinal and skin-related symptoms, including diarrhea, mucosal inflammation and rash, for PI3K pathway inhibitors. However, tolerance to these combinations warrants further evaluation.

## Conclusion

In summary, we investigated the significance of HMGA1 in iCCA and identified HMGA1 as an unfavorable prognostic biomarker. Our data are largely consistent with the literature and improved current understanding. Through RNA sequencing technologies, cancer data analysis, analysis of public CRISPR data, pharmacological profiling and related experimental verification, we provided compelling evidence that HMGA1 could induce CCND1 expression by promoting CCND1 transcription and activating the PI3K signaling pathway in iCCA. Next, the effect of palbociclib for monotherapy was limited due to acquired resistance in iCCA. Furthermore, increased cell cycle-related proteins and activation of the PI3K pathway in iCCA were associated with acquired palbociclib resistance, which PF-04691502 could reverse. Finally, we found that palbociclib could work synergistically with the PI3K/mTOR inhibitor PF-04691502 to induce tumor regression in HMGA1-driven iCCA preclinical models, suggesting that by combining FDA-approved drugs with minimal AEs, we could improve the treatment outcomes of this deadly malignancy. More importantly, our findings propose a new paradigm for the clinical treatment of iCCA, although further clinical investigations are warranted.

### Electronic supplementary material

Below is the link to the electronic supplementary material.

Supplementary Material 1

Supplementary Material 2

## Data Availability

All the data generated or analyzed in this study are included in this published article and its Additional files.

## Abbreviations

CCA: Cholangiocarcinoma
iCCA: Intrahepatic cholangiocarcinoma
TCGA: The Cancer Genome Atlas
GEO: Gene Expression Omnibus
GO: Gene Ontology
IC50: 50% inhibitory concentration
AEs: Adverse Events
