# Correction: HMGA1 augments palbociclib efficacy via PI3K/mTOR signaling in intrahepatic cholangiocarcinoma

**DOI:** 10.1186/s40364-024-00574-0

**Published:** 2024-02-16

**Authors:** Zhipeng Li, Huaxin Zhou, Zhijia Xia, Tong Xia, Gang Du, Strohmer Dorothee Franziska, Xiaoming Li, Xiangyu Zhai, Bin Jin

**Affiliations:** 1 Department of Hepatobiliary Surgery, The Second Hospital of Shandong University, Jinan, China; 2 The Second Clinical College of Shandong University, Jinan, China; 3 Department of General, Visceral, and Transplant Surgery, Ludwig-Maximilians-University Munich, Munich, Germany; 4 Organ Transplant Department, Qilu Hospital of Shandong University, Jinan, China

**Correction: Biomark Res 11, 33 (2023)**

**https://doi.org/10.1186/s40364-023-00473-w**

The authors found that an identical image (HUCCT1-Combine) was unintentionally overlaid in another region (HUCCT1-shHMGA1-1) of Fig. 6C, as a result of a malfunction in the AI import system (Adobe Illustrator CS5).

**Fig. 6:**
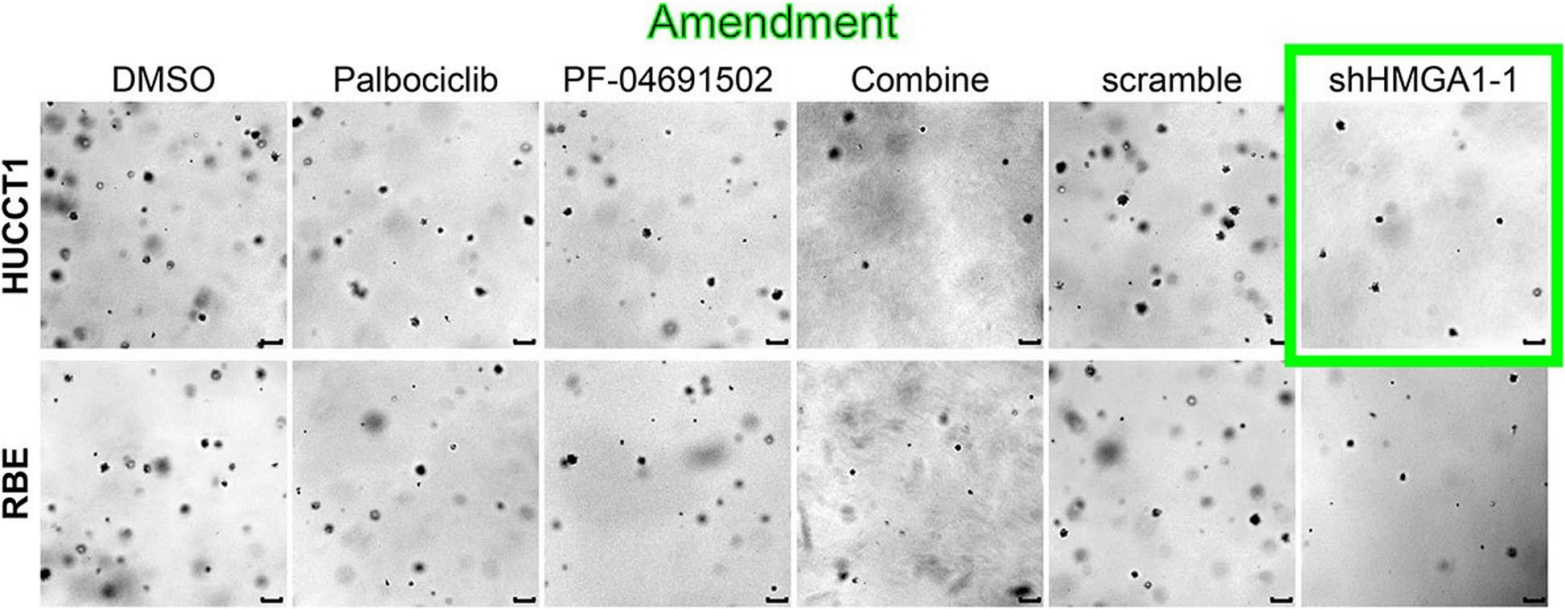
PF-04691502 works synergistically with palbociclib to inhibit iCCA growth, EMT and stemness in vitro. CCK − 8 assay (A), colony formation assay (B), 3D sphere formation assay (C), and transwell assay (D) analysis of iCCA cells treated with a single agent (PF-04691502 or palbociclib), a combination of both compounds at a fixed ratio (1:1) or shRNA-induced silencing of HMGA1. Analyzed data were from three independent experiments and shown as means ± SEM. Analysis for statistical significance was performed using Student’s t-test (n.s.,**, *** and **** represented not significant, *P* < 0.01, < 0.001and < 0.0001, respectively)

The authors wish to make the necessary replacement for the image in Fig. 6C (HUCCT1-shHMGA1-1) shown in this correction article [1].

All co-authors agree to the above revision.
