# Supplementary material for: HMGA1 augments palbociclib efficacy via PI3K/mTOR signaling in intrahepatic cholangiocarcinoma
Source: Biomark Res. 2023 Mar 29;11:33. doi: 10.1186/s40364-023-00473-w (PMC10053751; doi:10.1186/s40364-023-00473-w)
Supplement: Supplementary file 1 — Supplementary Material 1 [file 40364_2023_473_MOESM1_ESM.pdf]

Supplemental Figure S1. HMGA1 promoted iCCA progression.

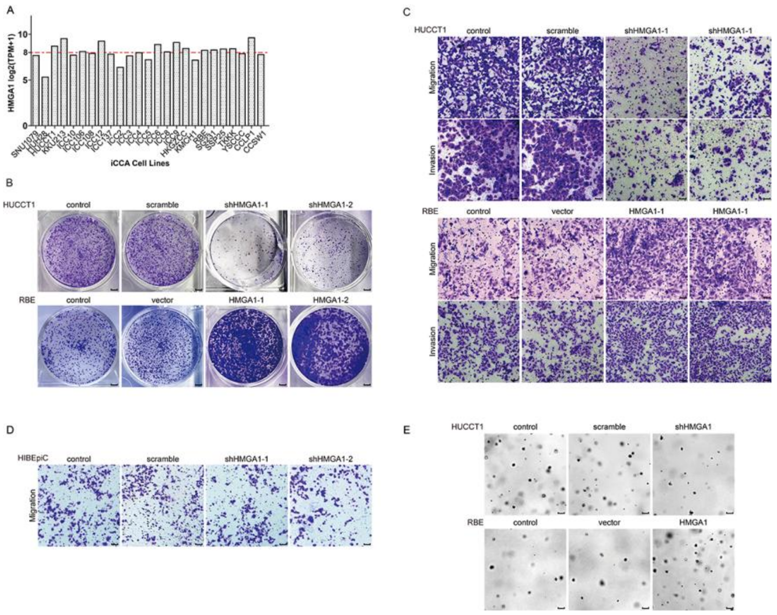

A, HMGA1 expression in ICC cell lines in the Broad Institute DepMap web portal.

B, Representative images of colony formation in Figure 1G.

C, Representative images of Transwell assay in Figure 1H,I. Scale bar: 50 μm.

D, Representative images of 3D cell sphere assay in Figure 1K. Scale bar: 200 μm.

Supplemental Figure S2. The correlations between HMGA1 and screened genes in TCGA and GEO database.

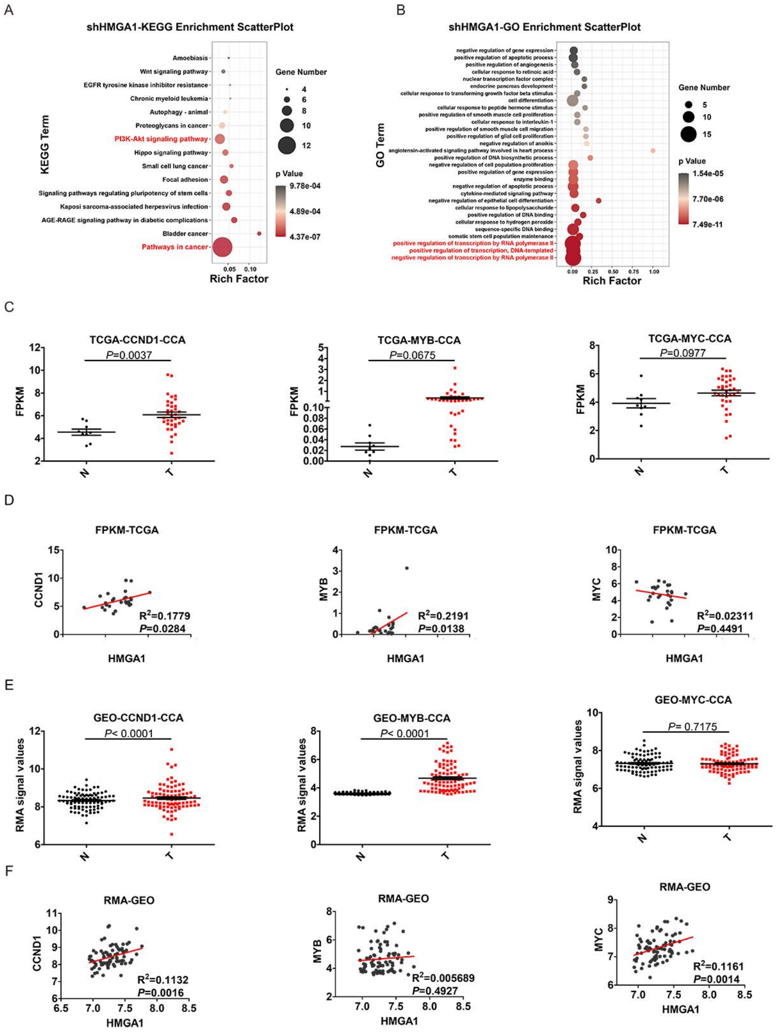

A-B, KEGG and GO enrichment of HMGA1 reduced signatures(HRSs).

C-F, Correlations between HMGA1 and 3 screened genes from the shHMGA1 gene expression profiling. The relevant data of genes expression were from TCGA and GEO database.

Supplemental Figure S3. CDK4/6 inhibitor emerge as strong dependencies across CCND1 relevant hepatobiliary cancers.

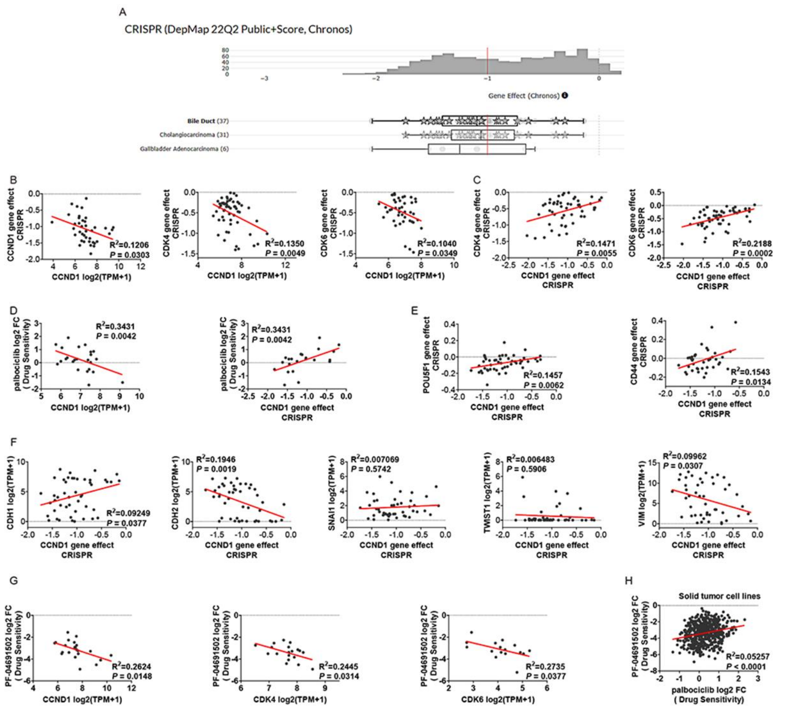

A, The gene effect of CCND1 in CCA.

B, Pearson correlation of CCND1 gene expression and CCND1/CDK4/CDK6 gene dependency in hepatobiliary cancer models.

C, Pearson correlation of CCND1 and CDK4/CDK6 gene dependency in hepatobiliary cancer models.

D, Comparison of CDK4/CDK6 inhibitor palbociclib drug sensitivity data from pharmacological dataset in hepatobiliary cancer models and CCND1 gene expression and CCND1gene dependency.

E, Pearson correlation of CCND1 and POU5F1/CD44 dependency in hepatobiliary cancer models.

F, Pearson correlation of CCND1 gene dependency and EMT-relevant genes expression in hepatobiliary cancer models.

G, Comparison of PI3K/mTOR inhibitor PF-04691502 drug sensitivity data from pharmacological dataset in hepatobiliary cancer models and CCND1/CDK4/CDK6 gene expression.

H, Comparison of CDK4/CDK6 inhibitor palbociclib and PI3K/mTOR inhibitor PF-04691502

drug sensitivity data from pharmacological dataset in solid tumor cell lines.

Supplemental Figure S4. palbociclib and PF-04691502 inhibited iCCA migration, invasion and stemness.

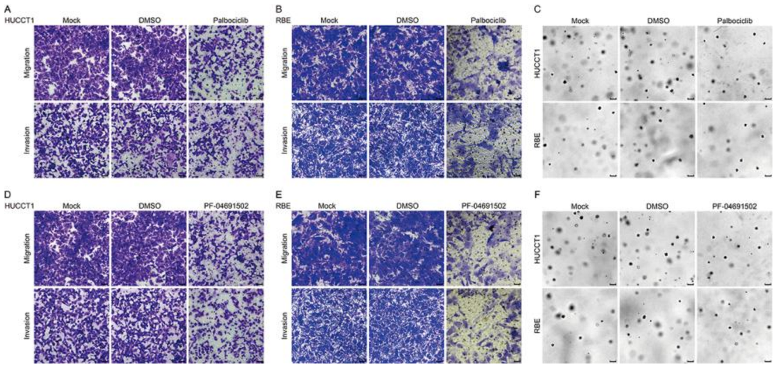

A-B, Representative images of Transwell assay in Figure 3E. Scale bar: 50 µm.

C, Representative images of 3D cell sphere assay in Figure 3H. Scale bar: 200 µm.

D-E, Representative images of Transwell assay in Figure 4D. Scale bar: 50 µm.

F, Representative images of 3D cell sphere assay in Figure 4G. Scale bar: 200 µm.

Supplemental Figure S5. PI3K-AKT and CDK4/6 pathway dual inhibition demonstrates efficacy in iCCA.

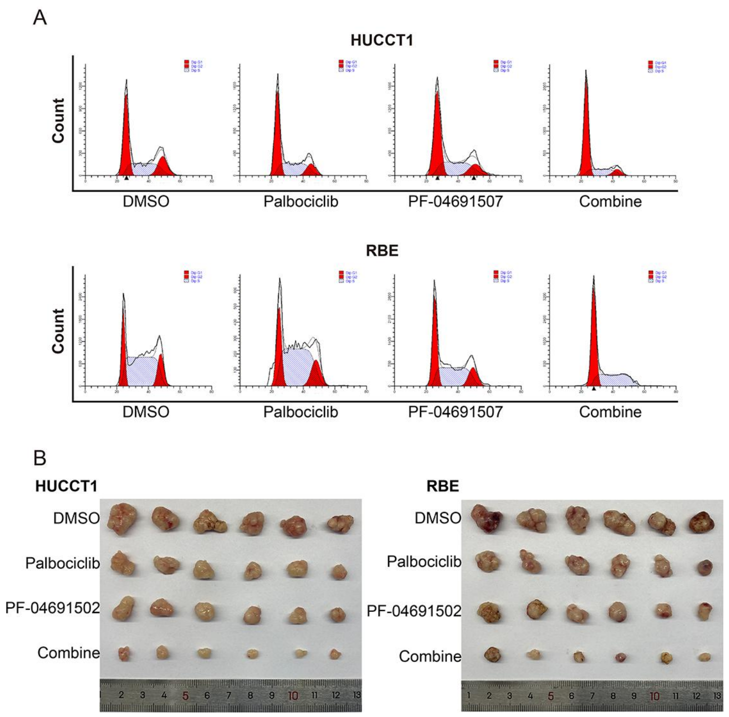

A, Representative images of cell cycle arrest in Figure 5A.

B, Representative images of xenografts tumors in Figure 7A.
