# Supplementary material for: HMGA1 augments palbociclib efficacy via PI3K/mTOR signaling in intrahepatic cholangiocarcinoma
Source: Biomark Res. 2023 Mar 29;11:33. doi: 10.1186/s40364-023-00473-w (PMC10053751; doi:10.1186/s40364-023-00473-w)
Supplement: Supplementary file 2 — Supplementary Material 2 [file 40364_2023_473_MOESM2_ESM.pdf]

Supplemental Table S1. The information of reagents and antibodies.

Reagen or resource	Source	Iden ififier
HMGA1	Abcam	Cat # ab129153
Cyclin-D1	Cell Signaling Technology	Cat # 55506
CDK4	Proteintech Group	Cat # 66950-1-lg
CDK6	Abcam	Cat # ab124821
OCT4	Proteintech Group	Cat # 60242-1-lg
CD44	Proteintech Group	Cat # 60224-1-lg
EMT antibodies kit	Cell Signaling Technology	Cat # 9782 T
RB	Proteintech Group	Cat # 67521-1-lg
p-RB	Cell Signaling Technology	Cat #8516S
PI3K	Cell Signaling Technology	Cat #84249T
p-PI3K	Cell Signaling Technology	Cat #4228S
AKT	Proteintech Group	Cat # 60203-2-lg
p-AKT	Proteintech Group	Cat # 66444-1-lg
mTOT	Proteintech Group	Cat # 66888-1-lg
p-mTOR	Proteintech Group	Cat # 67778-1-lg
PARP	Abcam	Cat # ab191217
C-PARP	Abcam	Cat # ab32064
β-actin	Cell Signaling Technology	Cat # 4970
GAPDH	Santa Cruz	Cat # sc-47724
740 Y-P	MedChemExpress	Cat # HY-P0175
Palbociclib	MedChemExpress	Cat # HY-50767
PF-04691502	MedChemExpress	Cat # HY-15177
CMC-Na	MedChemExpress	Cat # HY-Y0703

Supplemental Table S2 Primers for qPCR

Genes	Forward primer(5'-3')	Reverse primer(5'-3')
HMGA1	GGAAAAGGACGGCACTGAGA	TGGTGGTTTTCCGGGTCTTG
CCND1	GAAGGAGACCATCCCCCTGA	GAAATCGTGCGGGGTCATTG
EDN1	TCCCACAAAGGCAACAGACC	GCCCTGAGTTCTTTTCCTGCT
MYB	ATAGTCAATGTCCCTCAGCCAG	TTTCCTTCTCAGGGTCTTCATC
MYC	CCCCTACCCTCTCAACGACA	CTTCTTGTTCCTCCTCAGAGTCG
RUNX1	CCATCGCTTTCAAGGTGGT	CTCTTCCACTTCGACCGACA
ITGA6	GGAGGTACAGTTGTTGGCGA	TGCTGTGCCGAGGTTTGTAA
IL6	CAATGAGGAGACTTGCCTGGT	GCAGGAACTGGATCAGGACT
FGF2	AAGGAGTGTGTGCTAACCGT	CTGCCCAGTTCGTTTCAGTG
GAPDH	GAGTCAACGGATTTGGTCGT	GACAAGCTTCCCGTTCTCAG
OCT4	TCAGGAGATATGCAAAGCAGAA	TTGCCTCTCACTCGGTTCTC
CD44	CGTGGAGAAAAATGGTCGCT	TGTGGGCAAGGTGCTATTGAA

Supplementary Table S3 The sh/siRNA sequences

Name	The sh/siRNA sequences
shHMGA1-1	CAACTCCAGGAAGGAAACCAA
shHMGA1-2	ACCCCAGGAAGGAAACCAATT
Sh-scramble –HMGA1	TTCTCCGAACGTGTCACGT
SiCCND1-1	AACAAGCUCAAGUGGAACCUG
SiCCND1-2	CAGGUUCCACUUGAGCUUGUU
Scrambled CCND1	UUCUCCGAACGUGUCACGUTT

Supplementary Table S4 The promoter region sequences

Name	The promoter region sequence
HMGA1	GAAACCGAACGCCCTCCAGCCACCACCACTCACGGCCTACTAAGCGCGCGCT<br>CCGGGCCCAGGGCACAAAGATGGAGGGAGCGGCGCGATCCACCCGCCCCACC<br>CCCACCCACCAGACAAAGGGGCAGGCCGCCCCTCCCCCAGCCTTCTTCGCGG<br>GCCCCGCCGCCCTCTGCTCCCCTCCCCTCGGGCCGCCCTTTGGTGACTTCCTTT<br>CCCCTCCAAGGGCGGCCCCGGGACTTCTCAGGAACTGCGTTTCACCTGGACG<br>TGGGGCGGGGAGCCGGCGCGCAGAGGGCGGCCTCCGGGCCCACCCCTAGGC<br>CGAGCCGGCCCCAGGCCCGCGCCCTCCCCTTTGAACCCGCGCTCCCGGCCCCT<br>GGCGCCTTCCCGCCCAGGCGCCCCCGCCCCCACCCGGTCAAGCACGTGCTGC<br>CCGGGCCCCGAGCGCTTCCCGCCGCACGGGTGGGGGCTGGGCCCACCCCCCG<br>CCCGCTCCCCACCCCCACCGGAAACATTCCTGCCACATTCCTGCAACTGCAAG<br>GCCCAGCCCGCCGCTCCCCACCCCGCCTCCGCGCCGGGCCCAGGGCTTTCCTG<br>CGTCCCCTCCACCGCCGGCTCGCCCCCTGAGGAGGGGGCTGGGCCAGGGCCT<br>CGGCTGACCGGGGAGGAAGAAGGGGAGCAGAGAAAAACATGAGTCACAGCC<br>GTGTGTCACTGGAGCGCATTTCAATTCCCTGCATCACAGGAGGTGTGGAAGGC<br>CGCCTCGGGGACCGGGCGCGGGAGGTGCGCCCGAGAAGGCCCCGGGCCGGC<br>CTGCAGGGCGCGCCGCTCCGCCTGCGCCCTTTCCTCCCCCACCGCCCTCCCCG<br>CCATCTTCCCCTTTGGCTTCCTTCTCGCTCGGTGCAACAAGTCTTTGTTAAGCC<br>GGGCGCCGGGCGGGCCAGGCGTGGCGGAGATGGCCTGCGTGCTCGGCCCCTG<br>CCCTCAAAGCGCTTCCAGGCGACCTCTGGCCCACCTTTTATTTTTATTTACAGT<br>CCCAACGGAAGGGCCCAGGTCCCCAAGTGGGCCTGCGTATCTCCAGAACACC<br>ATCTAAGTCACCTCAAGGTATGAAGCCTCCCTTGGGTGTACCTGCCAACGAGC<br>CAATCGTTGGTTTCGCTGGAAGGCTCCACCTTGATCATGGCTCGCTGGTGGCC<br>ATTAATAAAACACTTTGGATTTCACAAGTTTCACGTTTGAATTTCACAAGACTT<br>GTATCTCACCAATCAGCCACACGCGGGTTGACACTGAAAGGCACATGCTGACA<br>CCTGTCCGCCCAGGGGAAATCTACCATCTCTCTATTTTAGTTAGGGGGCGGCGT<br>GTAGGCCGCGGAATTCTCTAACGAGCGCGTTTCTCTTCACCCCCTGGGCCCGC<br>GTGGACCCCCGTCCACCCCCACACGCCCTGGGGGGGGGCCGGGCCCACACG<br>CCCTGGAAGCCCCTAGAGGTGACTCTCCCTGGGACCCCTGTACCAGGGAGGA<br>AGGATACCGCCACCCGTCACCACCCCCCGCCAGAAGCTCCTTCGTGACTCCTC<br>TGCGCGTGCCTTCCCACACCTCCTCCGTCCGGGACTGCGAGGAGTGGGCGGT<br>CGACTCGAGTTCGCAGCCCAGGCCTCCCACACGCCCCTCCCTGCCGTCAGCAC<br>CCACCCGCGCGGCAGCGGCGGCGGCGGCTGGCGGGCGGCCGCCCTTTTAAAT<br>CCCCGGGCTCATTTGCATGGCCCCGCCCCCTGAGTGACACGGCTGGCGCGGGC<br>GGGCCCGTCCCCCCTGCCCCTGGGTCGCTCTTTTTAAGCTCCCCTGAGCCGGT<br>GCTGCGCTCCTCTAATTGGGACTCCGAGCCGGGGCTATTTCTGGCGCTGGCGC<br>GGCTCCAAGAAGGCGTGAGTTCGCGGCCGCTCCGGTGGCTTCTTTTTTTTATAT<br>CTATAATTTAATTAAATTATTTATTTATTGAGGCCGCGCACGGGCCGTGCCCAGC<br>TTCCTGCCCCTCGCCATCCTTC

CCND1	TGTCTTGCGCCCGGGATGGGGGGGTGAAGCTCCCTCCTGGACCCAGAGCCGG<br>TTGTGCCGGAGTGGGCGAGCCTCTTTATGCCCTGCTGCCCCTAGCCGACTTCG<br>GCCCGCTTCGCGCCTCGGGCTGGGCCAGGGCGCACGCGGGGCTCGGGGCCCC<br>TCGCCCCACGGGATGGGAGAGGCCGGGTGATAGCTCCGGGCCCCATAAATCAT<br>CCAGGCGGCCGCCGGGTCGGGATTTTATGAATGAAAAAGCAGCTGGGCCGCC<br>CTTGTGCGCGGGCTGATGCTCTGAGGCTTGGCTATGCGGGGGCCAACGCGATT<br>GTGGGTGCTCGGGGAGTGGGGGGGGGCACGACCGTAGGTGCTCCCTGCTGGG<br>GCAACCCATCGCTCCCCATGCGGAATCCGGGGGTAATTACCCCCCCAGGACCC<br>GGAATATTAGTAATCCTAATTCCCGGCGGGGGAGGGGGCGCGGGAGGAATTCA<br>CCCTGAAAGGTGGGGGTGGGGGGGGTCGCATCTTGCTGTGAGCACCCTGGCG<br>AAGGGGAGAGGGCTTTTTCTATCAGTTTTCTTTGAGCTTTTACTGTTAAGAGGG<br>TACGGTGGTTTGATGACACTGAACTATATTCAAAAGGAAGTAAATGAACAG<br>TTTTCTTAATTTGGGGCAGGTACTGTAAAAATAAAAACAAAAGTTAAGACAGT<br>AAAATGTCCTTTTATTTTTTAATGCACCAAAGAGACAGAACCTGTAATTTTAAA<br>AACTGTGTATTTTAATTTACATCTGCTTAAGTTTGCGATAATATTGGGGACCCTC<br>TCATGTAACCACGAACACCTATCGATTTTGCTAAAAATCAGATCAGTACACTCG<br>TTTGTTTAATTGATAATTGTTCTGAATTATGCCGGCTCCTGCCAGCCCCCTCACG<br>CTCACGAATTCAGTCCCAGGGCAAATTCTAAAGGTGAAGGGACGTCTACACCC<br>CCAACAAAACCAATTAGGAACCTTCGGTGGTCTTGTCCCAGGCAGAGGGGAC<br>TAATATTTCCAGCAATTTAATTTCTTTTTTAATTAAAAAAAATGAGTCAGAATGG<br>AGATCACTGTTTCTCAGCTTTCCATTCAGAGGTGTGTTTCTCCCGGTTAAATTG<br>CCGGCACGGGAAGGGAGGGGGTGCAGTTGGGGACCCCCGCAAGGACCGACT<br>GGTCAAGGTAGGAAGGCAGCCCGAAGAGTCTCCAGGCTAGAAGGACAAGAT<br>GAAGGAAATGCTGGCCACCATCTTGGGCTGCTGCTGGAATTTTCGGGCATTTAT<br>TTTATTTTATTTTTTGAGCGAGCGCATGCTAAGCTGAAATCCCTTTAACTTTTAG<br>GGTTACCCCCTTGGGCATTTGCAACGACGCCCCTGTGCGCCGGAATGAAACTT<br>GCACAGGGGTTGTGTGCCCGGTCCTCCCCGTCCTTGCATGCTAAATTAGTTCTT<br>GCAATTTACACGTGTTAATGAAAATGAAAGAAGATGCAGTCGCTGAGATTCTT<br>TGGCCGTCTGTCCGCCCGTGGGTGCCCTCGTGGCGTTCTTGGAAATGCGCCCA<br>TTCTGCCGGCTTGGATATGGGGTGTCGCCGCGCCCCAGTCACCCCTTCTCGTG<br>GTCTCCCCAGGCTGCGTGTGGCCTGCCGGCCTTCCTAGTTGTCCCCTACTGCA<br>GAGCCACCTCCACCTCACCCCCTAAATCCCGGGGGACCCACTCGAGGCGGAC<br>GGGGCCCCCTGCACCCCTCTTCCCTGGCGGGGAGAAAGGCTGCAGCGGGGCG<br>ATTTGCATTTCTATGAAAACCGGACTACAGGGGCAACTCCGCCGCAGGGCAGG<br>CGCGGCGCCTCAGGGATGGCTTTTGGGCTCTGCCCCTCGCTGCTCCCGGCGTT<br>TGGCGCCCGCGCCCCCTCCCCCTGCGCCCGCCCCCGCCCCCCTCCCGCTCCCA<br>TTCTCTGCCGGGCTTTGATCTTTGCTTAACAACAGTAACGTCACACGGACTA<br>CAGGGGAGTTTTGTTGAAGTTGCAAAGTCCTGGAGCCTCC
